# Hospital Expenditure.—The Commissariat

**Published:** 1896-09-19

**Authors:** 


### The Institutional Workshop.

### HOSPITAL EXPENDITURE.—THE COMMISSARIAT.

### XXVII.—THE COST OF BREAD.

The stability of this article of expenditure, which varies wonderfully little from one hospital to another in town and country, may be accounted for by the fact that the price paid is substantially the same in all localities, and that the quantity required by each member of the household, whether patient, doctor, nurse, or servant, is almost identical.

| London Hospitals. | Average yearly cost of each inmate for bread, &c. £ s. d. |
|---|---|
| St. Thomas's | 0 16 0 |
| University | 0 17 0 |
| Guy's (private bakehouse) | 1 0 0 |
| Great Northern Central | 1 0 0 |
| Middlesex | 1 0 0 |
| Westminster | 1 0 0 |
| Metropolitan | 1 0 0 |
| Royal Free | 1 2 0 |
| St. George's | 1 3 0 |
| St. Mary's | 1 3 0 |
| Charing Cross | 1 4 0 |
| Seamen's | 1 9 0 |
| London Hospital Nursing Home | 1 10 0 |

From the above table it will be seen that the lowest averages are at St. Thomas's and University, where few besides patients are boarded under the general account. At the Seamen's, where the average is highest, nearly all the patients are men under surgical treatment with hearty convalescent appetites, and the regular allowance of bread for full diet is 1 lb. a day. Now at 4d. per quartern loaf, each person consuming 1 lb. of bread a day cost 30s. a year.

Even reckoning for each member of the household alike to eat the regulation 12 ozs. of bread, the annual cost for each would not be under £1 3s., so that the above figures seem conclusively to prove that no leakage occurs under this heading in any of the hospitals named. The averages at provincial institutions are much the same:

| | £ s. d. |
|---|---|
| Leeds Gen. Infirm. (private bakehouse) | 0 16 0 |
| Bradford (private bakehouse) | 0 17 0 |
| Hull | 0 17 0 |
| York County (private bakehouse) | 0 18 0 |
| Birmingham, Queen's | 0 18 0 |
| Bristol General | 0 19 0 |
| Radcliffe, Oxford | 1 0 0 |
| Norfolk and Norwich | 1 2 0 |
| Halifax | 1 2 0 |
| Newcastle-on-Tyne | 1 2 0 |
| Sheffield General Infirmary | 1 2 0 |
| Birmingham General | 1 4 0 |
| Sussex County | 1 4 0 |
| Cambridge, Addenbrooke's | 1 6 0 |
| Liverpool Royal Infirmary | 1 8 0 |
| Ayr County | 1 0 0 |
| Edinburgh Royal Infirmary | 1 1 0 |
| Aberdeen | 1 2 0 |
| Glasgow Royal | 1 4 0 |
| Glasgow Western | 1 5 0 |
| Belfast Royal | 1 6 0 |

It must not be forgotten in studying the above table that several other items are included in the uniform system of accounts under the heading "Bread." These include flour, oatmeal, biscuit (used in the North), barley, diabetic foods, cakes, and confectionery. Flour appears to be comparatively little used in London hospitals, but in some parts of the country it forms an important item. Scotch hospitals and others in the north of England use a good deal of oatmeal, the yearly outlay on flour and oatmeal varying from 2s. a head at Aberdeen, to 5s. at the Edinburgh Royal and Glasgow Royal Infirmaries, and at the Glasgow Western rising to 7s. a head. In the Belfast Royal the yearly outlay on barley and oatmeal amounts to 2s. a head. It is impossible not to regret the disuse of oatmeal at the London and other southern county hospitals. Difficult as the London patient is to reconcile to any unfamiliar article of diet, few would be found to despise a comfortable bowl of well-made milk gruel for supper, the meagreness of which meal is a standing grievance in some hospitals, and a fruitful source of wakeful nights.

It may be interesting to see the exact amount of bread consumed per head in the year at the following institutions:—

| | |
|---|---|
| Bristol General Infirmary | 219 lbs. |
| Sussex County | 245 lbs. |
| Oxford, Radcliffe | 263 lbs. |
| Belfast Royal | 294 lbs. |
| Norfolk and Norwich | 295 lbs. |

The amount consumed at Guy's was 239 lbs. for each patient (or rather each occupied bed), and 245 lbs. each for other members of the household. In the Birmingham General the patients consumed 224 lbs. a head in the year, and the rest of the household only 162 lbs. each. The latter amount is so small as to suggest the probability that home-made bakings of scones, or other bread-stuffs, take the place of ordinary bread at some meals. It would appear from these figures that 12 oz. per day, or 273 lbs. each a year is a very fair average for the hospital household taken all together.

We have said that the price of bread differs little in various localities. A penny a pound, or 9s. 4d. per cwt. is the common London price. In two hospitals 11s. per cwt., or rather under 1½d. a pound, is paid, and in one the price sinks to 3¾d. a quartern, or 8s. 9d. the cwt.

In one or two provincial hospitals we note the pernicious custom of two qualities of bread—one for officials, the other for patients—a distinction for which in respect to bread there can be no excuse. There is more variation of price out of London. At Bristol the quartern loaf sinks to 3d., or 7s. per cwt.; at Oxford and Hull it is 3¼d., and at Norwich 3¾d. It rises to 4¼d. at Brighton, and 4½d. per quartern at Birmingham, Leeds, Glasgow, and Edinburgh.

It will be noticed that the lowest averages for bread occur where the baking is done on the premises, viz., at the Leeds and Bradford Infirmaries, and York County Hospital. Hull Infirmary runs these very close however. Where the necessary space can be had, and where the quantity of bread required is large enough to cover the wages of the baker, there can be no doubt that a considerable saving can be effected by home baking. Every shilling a-head saved reckons for over £25 a year in an institution the size of the Leeds Infirmary. It is not always easy to compute the cost of the bakery, including, as it should do under the heading of bread, the following items:—

(a) Extra fuel used in bakehouse.

(b) Wages of baker, and if boarded, allowance for board.

(c) Flour.

(d) Yeast, salt, &c.

At the Marylebone Infirmary, where the inmates number over a thousand, and things can be done on a large scale, the cost of the quartern loaf baked on the premises works out at 2½d.

In smaller institutions the principal saving is in such articles as diabetic foods, the cost of making which is little over half the shop price. At the Radcliffe Infirmary, where no other baking is done, the gluten bread is made on the premises, and with a little management this plan might be followed with great advantage in many institutions where these costly foods are a heavy extra expense.

The only London hospital where baking is undertaken is Guy's, where the diabetic preparations are a speciality and of the finest quality.

In addition to the saving effected by the private bakehouse, there can be no doubt that when it is properly conducted the bread is far superior in quality to that which can be bought, and, moreover, an appetising variety of bread-stuffs can be provided at the smallest cost.

The hospital bakehouse would, however, in many cases add only one more additional burden to shoulders already too well laden, and it is never likely to come into general use.

